# Supplementary material for: Quantifying the spatiotemporal mechanical dynamics of engineered cardiac microbundles
Source: PLoS Comput Biol. 2026 Jul 20;22(7):e1014522. doi: 10.1371/journal.pcbi.1014522 (PMC13492994; doi:10.1371/journal.pcbi.1014522)
Supplement: Supplementary file 1 [file pcbi.1014522.s001.pdf]

Supporting Information: Supplementary Figure 1

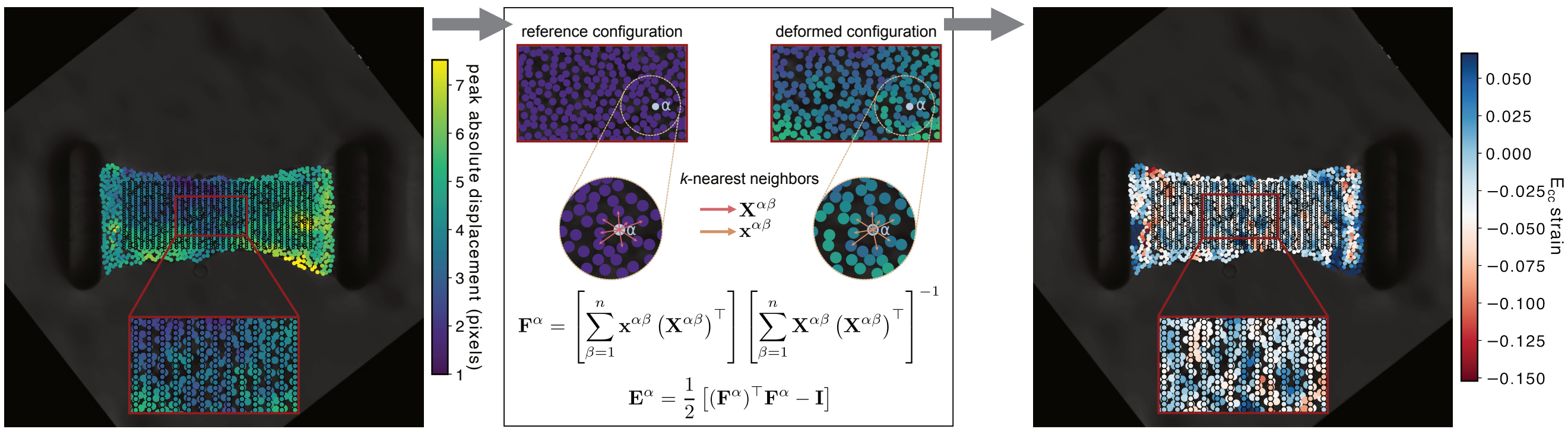

Fig S1. Details of computing a Green-Lagrange strain field from tracked displacement data. The left panel shows the absolute displacement field at peak contraction, obtained at the tracked fiducial marker points and interpolated onto a 28×33 regular grid; the zoomed-in inset highlights the spatial distribution of fiducial marker points within the tissue domain. Directional displacement fields in the column (horizontal) and row (vertical) directions are obtained analogously. The locations of the tracked fiducial marker points in both the reference (undeformed) and current (deformed) configurations serve as the input to the strain computation illustrated in the middle panel. There, a k-nearest neighbor approach is used to estimate the local deformation gradient at each fiducial marker point. For a given marker point α (here shown with $k = 8$ neighbors), the vectors $\mathbf{X}^{\alpha\beta}$ and $\mathbf{x}^{\alpha\beta}$ connect point α to each neighbor β in the reference and current configurations, respectively. The deformation gradient tensor $\mathbf{F}^{\alpha}$ is estimated from these neighbor vectors via a least-squares fitting procedure, and the Green-Lagrange strain tensor $\mathbf{E}^{\alpha}$ is subsequently computed as shown. Repeating this procedure at every fiducial marker point yields the full-field strain estimate shown in the right panel, where the Green-Lagrange strain in the column-column direction E_{cc} is displayed at all fiducial marker points and interpolated onto the 28×33 regular grid. Positive and negative values indicate local extension and compression, respectively, as indicated by the color scale.
