# Supplementary material for: Quantifying the spatiotemporal mechanical dynamics of engineered cardiac microbundles
Source: PLoS Comput Biol. 2026 Jul 20;22(7):e1014522. doi: 10.1371/journal.pcbi.1014522 (PMC13492994; doi:10.1371/journal.pcbi.1014522)
Supplement: Supplementary file 2 [file pcbi.1014522.s002.pdf]

Supporting Information: Supplementary Figure 2

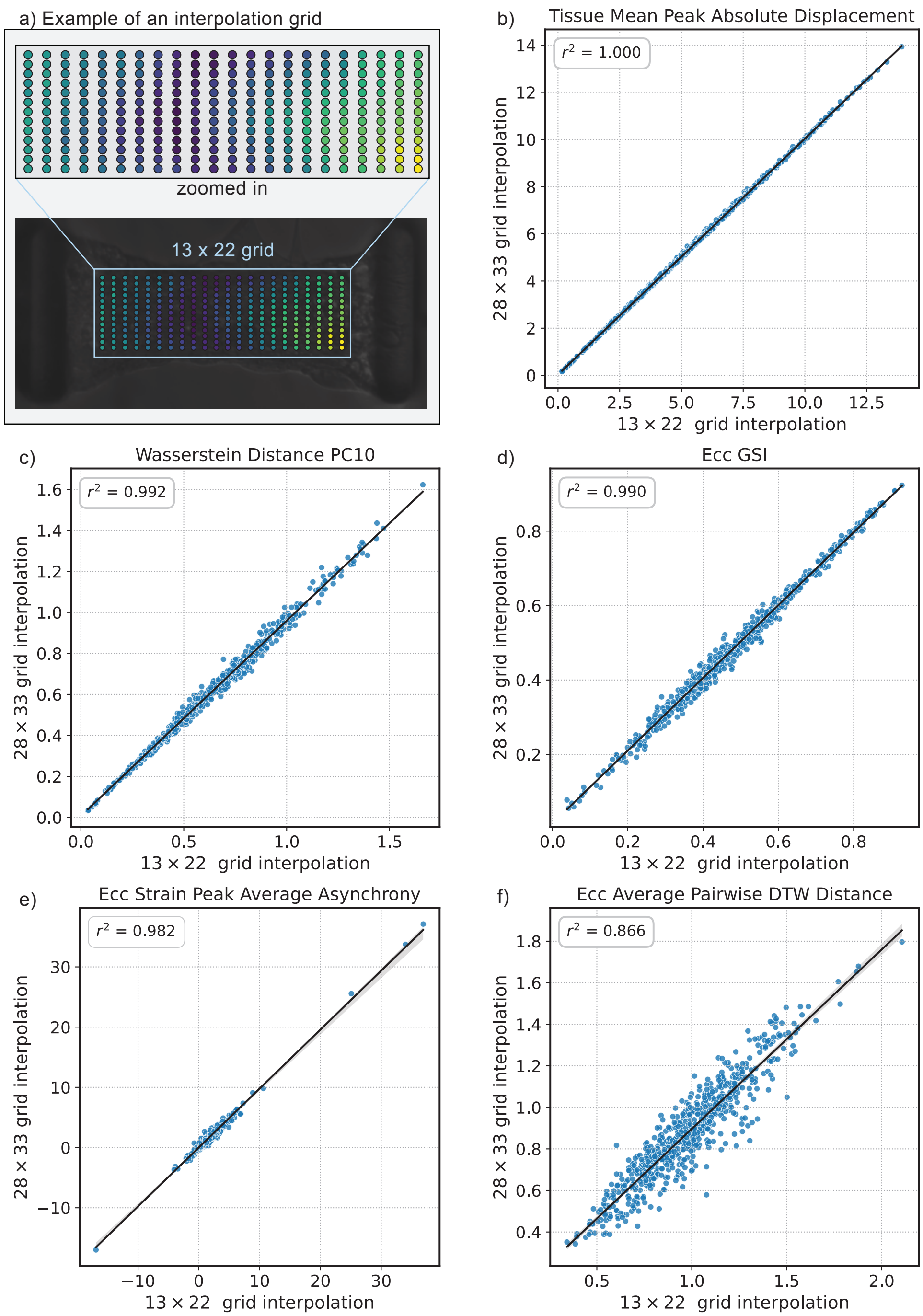

Fig S2. Grid sensitivity analysis for all grid-dependent metrics. (a) Example of a tissue-specific 13×22 interpolation grid used for displacement and strain calculations, with a zoomed-in view. For our main analysis, a finer 28×33 grid is applied. Subplots (b–f) compare metric values computed on both grid resolutions, demonstrating convergence with increasing grid size. While most metrics show strong agreement (high r^2 values) between grids, "E_{cc} Average Pairwise DTW Distance" exhibits greater sensitivity to grid resolution. Based on these results, the 28×33 grid provides sufficient resolution for robust metric calculations.
