# Supplementary material for: Quantifying the spatiotemporal mechanical dynamics of engineered cardiac microbundles
Source: PLoS Comput Biol. 2026 Jul 20;22(7):e1014522. doi: 10.1371/journal.pcbi.1014522 (PMC13492994; doi:10.1371/journal.pcbi.1014522)
Supplement: Supplementary file 3 [file pcbi.1014522.s003.pdf]

Supporting Information: Supplementary Figure 3

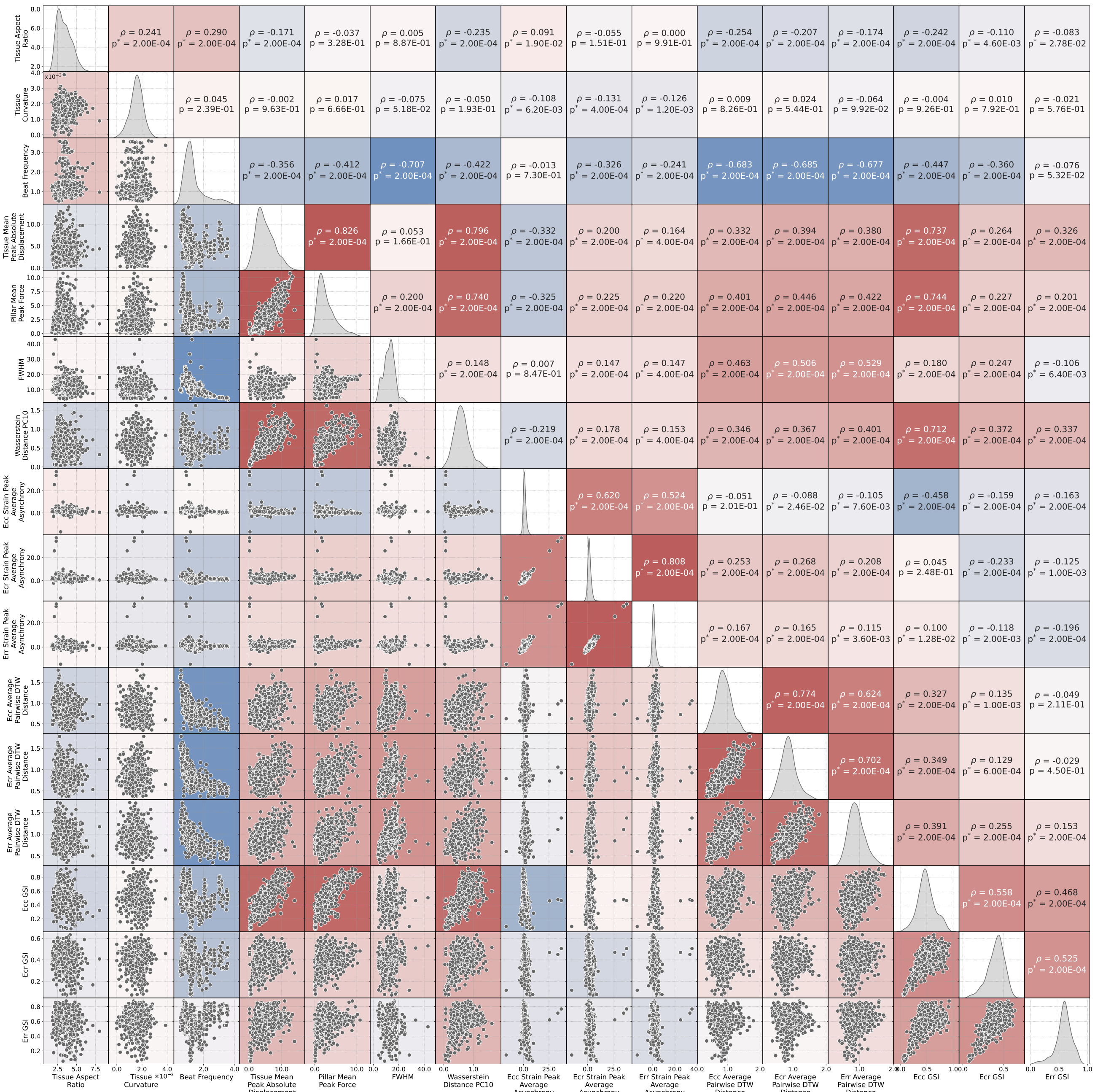

Fig S3. Expanded version of Fig. 10 displaying all 16 extracted metrics. The upper triangle presents the Spearman's correlation coefficients (ρ) for each metric pair, accompanied by their respective p-values. The lower triangle contains pairplots depicting the joint distributions for each pair of metrics, providing further support for the monotonicity assumption underlying the Spearman correlation analysis. Along the diagonal, Kernel Density Estimate (KDE) plots illustrate the individual distribution of each metric, highlighting differences in data spread and underlying variability across metrics.
