# Supplementary material for: Quantifying the spatiotemporal mechanical dynamics of engineered cardiac microbundles
Source: PLoS Comput Biol. 2026 Jul 20;22(7):e1014522. doi: 10.1371/journal.pcbi.1014522 (PMC13492994; doi:10.1371/journal.pcbi.1014522)
Supplement: Supplementary file 4 [file pcbi.1014522.s004.pdf]

# **Supporting Information:** Supplementary Figure 4

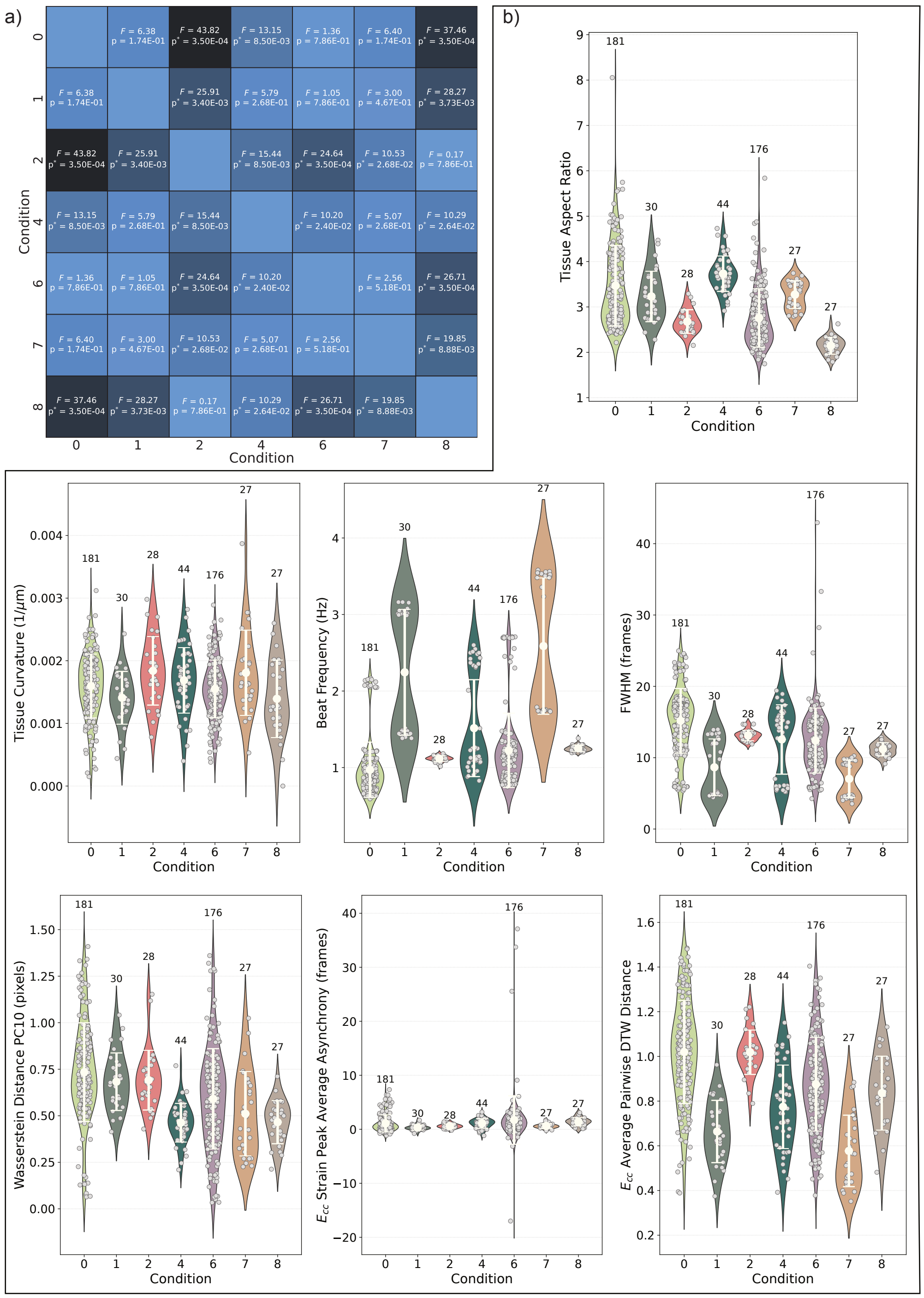


**Fig S4. Multivariate statistical analysis across experimental conditions.** (a) Annotated heatmap of PERMDISP results, where deeper blue tones represent higher pseudo-$F$ statistics. P-values are shown for each condition pair, with an asterisk indicating statistical significance. (b) Violin plots for the remaining 7 metrics from Fig. 13, depicting the distribution, mean, and standard deviation for each condition.
