# Supplementary material for: Quantifying the spatiotemporal mechanical dynamics of engineered cardiac microbundles
Source: PLoS Comput Biol. 2026 Jul 20;22(7):e1014522. doi: 10.1371/journal.pcbi.1014522 (PMC13492994; doi:10.1371/journal.pcbi.1014522)
Supplement: Supplementary file 5 [file pcbi.1014522.s006.pdf]

# Quantifying the Spatiotemporal Dynamics of Engineered Cardiac Microbundles - [PCOMPBIOL-D-26-00827]
## Response to Reviewers

We would like to begin by sincerely thanking the Editor and the Reviewers for the time, effort, and thoughtful consideration they devoted to evaluating our manuscript. We greatly appreciate their constructive feedback and the opportunity to further strengthen this work.

In response to the reviewers' comments, we have carefully revised the manuscript and implemented a number of changes aimed at improving its clarity, rigor, and overall impact. These revisions include clarifications to the text, updates to several figures and figure captions to better explain the methods, and additions that strengthen both the discussion and the presentation of future research directions. We have also added a new supplementary figure to further illustrate a key methodological component of the study.

We believe that these revisions have substantially improved the manuscript, and we are grateful for the reviewers' insights, which helped us refine both the presentation and interpretation of our work. All modifications are highlighted in red in the revised submission. Our detailed point-by-point responses to each comment are provided below.

**Academic Editor Comments and Response**

1. Please ensure that the CRediT author contributions listed for every co-author are completed accurately and in full. At this stage, the following Authors/Authors require contributions: Brendon M. Baker, Samuel J. DePalma, Javiera Jilberto, Hiba Kobeissi, David Nordsletten, and Emma Lejeune. Please ensure that the full contributions of each author are acknowledged in the "Add/Edit/Remove Authors" section of our submission form. The list of CRediT author contributions may be found here: `https://journals.plos.org/ploscompbiol/s/authorship#loc-author-contributions`

   - Thank you for pointing this out and sharing the relevant link. We have completed the list of author contributions.

2. We ask that a manuscript source file is provided at Revision. Please upload your manuscript file as a .doc, .docx, .rtf or .tex. If you are providing a .tex file, please upload it under the item type u2018LaTeX Source Fileu2019 and leave your .pdf version as the item type u2018Manuscriptu2019.

   - We have uploaded the .tex source file of the revised manuscript in addition to the .pdf version.

3. Your manuscript's sections are not in the correct order. Please amend to the following order: Abstract, Introduction, Results, Discussion, and Methods.

   - Thank you for raising this point. From the PLOS Computational Biology author guidelines we see the statement: "To provide flexibility, however, authors are also able to include the Materials and Methods section before the Results section or before the Discussion section. Please clarify the reasons for including your Materials and Methods section before the Results or Discussion sections in your cover letter when submitting your manuscript files." Sincere apologies for not justifying the choice in our cover letter! In the revised submission we have addressed this. Should the editor find it acceptable, we respectfully request to retain the existing section order.

4. Please upload all main figures as separate Figure files in .tif or .eps format. For more information about how to convert and format your figure files please see our guidelines: `https://journals.plos.org/ploscompbiol/s/figures`

- Thank you for sharing this helpful resource. We have submitted the figures in .eps format as requested.

5. We notice that your supplementary Figures are included in the manuscript file. Please remove them and upload them with the file type 'Supporting Information.' Please ensure that each Supporting Information file has a legend listed in the manuscript after the references list.

   - Thank you for bringing this to our attention. We have removed the supplementary figures from the manuscript file and uploaded them separately as 'Supporting Information.' We have also included the corresponding captions and legends in the manuscript after the references list.

6. Please amend your detailed Financial Disclosure statement. This is published with the article. It must therefore be completed in full sentences and contain the exact wording you wish to be published. 1) State the initials, alongside each funding source, of each author to receive each grant. For example: "This work was supported by the National Institutes of Health (####### to AM; ###### to CJ) and the National Science Foundation (###### to AM)." 2) State what role the funders took in the study. If the funders had no role in your study, please state: "The funders had no role in study design, data collection and analysis, decision to publish, or preparation of the manuscript." 3) If any authors received a salary from any of your funders, please state which authors and which funders. If you did not receive any funding for this study, please simply state: u201cThe authors received no specific funding for this work.u201d.

   - Thank you for these detailed guidelines. We have revised the Financial Disclosure statement to be in accordance with the specified requirements.

---

**Reviewer 1 Comments and Response**

---

1. It's not really clear how this method translates from 2D to 3D systems. Has there been any validation to show that what's measured in 2D holds up in more physiologically relevant 3D tissues?

   - We thank the reviewer for this thoughtful comment and agree that the relationship between the present 2D image-based analysis and more physiologically relevant 3D engineered tissues warrants clarification. We interpret the comment as raising two related issues: whether the tissue platforms themselves are representative of 3D systems, and whether the image-based analysis framework can be extended from 2D time-lapse imaging to fully 3D spatiotemporal imaging.
   - With respect to the tissue platforms, the fibroTUG tissues analyzed here are not conventional 2D monolayers; they are formed from electrospun fiber matrices suspended between flexible posts and are therefore more appropriately described as quasi-3D or 2.5D engineered tissues. In addition, the core tracking tools used in this study, "MicroBundleCompute" and "MicroBundlePillarTrack," have previously been implemented and validated on standard cardiac microbundles, which are self-assembled 3D tissues suspended between posts. We have clarified this point in the revised manuscript and directed readers to the relevant prior publications where these tracking and force-quantification approaches were validated.
   - With respect to the imaging framework, we acknowledge that the present study analyzes 2D time-lapse image sequences and therefore quantifies projected in-plane displacement and strain rather than full 3D deformation. Some elements of the framework (including registration, displacement tracking, strain-based synchrony metrics, and dimensionality reduction) are conceptually extensible to 3D volumetric time-lapse data, but other components would require methodological adaptation, including 3D strain reconstruction and extension of critical point analysis from 2D to 3D vector-field topology. We have revised the manuscript to make this limitation explicit and to avoid implying that the current implementation directly provides full 3D mechanical characterization.

- Because high-resolution volumetric imaging with sufficient temporal resolution to capture beating engineered cardiac tissues remains technically challenging and is not yet routine for high-throughput studies, the present framework is intended as a scalable and interpretable approach for 2D time-lapse imaging datasets, including projected measurements from quasi-3D and 3D microtissue platforms. As volumetric imaging technologies continue to mature, we hope the principles introduced here will serve as a useful foundation for future extensions to fully 3D spatiotemporal mechanical analysis.
- The relevant changes are highlighted in red in the "Time-lapse image data extraction" section and in the "Conclusion" section. Thank you for this comment, as we believe these clarifications meaningfully strengthen the manuscript.

2. The scalability across different tissue platforms feels underdeveloped. Tissue sizes vary a lot, and so do imaging setups. Right now, it's not clear at what resolution this method starts to break down. There's no guidance on minimum pixel size, no sensitivity analysis for resolution, and no real discussion of how tracking accuracy changes as resolution drops.

    - We thank the reviewer for raising this point. Similar to our response to the previous comment, we would like to direct the reviewer to our prior publications, which were entirely focused on validating the tracking frameworks [1, 2]. In both prior publications, the tracking approaches were validated across both the FibroTUG platforms and the more common 3D cardiac microbundle platforms. We emphasize that the scope of the present paper is not to elaborate on the tracking software itself, but rather to discuss the downstream analysis that is enabled by access to full-field tracking data.
    - For reference, the real-world datasets analyzed using "MicroBundleCompute" and "MicroBundlePillarTrack" include microbundles with spatial resolutions of 4 $\mu m$/pixel and 0.908 $\mu m$/pixel, beating frequencies ranging from $\sim 0.2$ Hz to $\sim 1.65$ Hz, and a mix of brightfield and phase-contrast imaging modalities. While an exhaustive study on the upper and lower bounds of (inter-dependent and likely platform specific) trackable spatial resolution and beating frequency has not been conducted, no instances of pipeline failure attributable to these factors have been encountered. Failure cases that have been observed are associated with low image brightness and/or contrast, as discussed in the "Software failure and data exclusion" section. Furthermore, in the work introducing "MicroBundleCompute" [1], the degradation of tracking accuracy is examined by introducing synthetic artifacts simulated as Perlin noise into the time-lapse image sequences.
    - To eliminate ambiguity regarding image resolution consistency within the fibroTUG dataset, we note that all images were acquired at a uniform resolution of 0.908 $\mu m$/pixel. The "Dataset" section in the revised manuscript has been updated to include this information, with the relevant changes highlighted in red. We thank the reviewer for prompting us to make these details more explicit.

3. FWHM is introduced without explanation (Section 2.2.1) and only defined later. It would help to define it right when it first appears.

    - Thank you for pointing this out. FWHM has now been defined at its first appearance in the "MicroBundlePillarTrack" section, with the relevant changes highlighted in red in the revised manuscript.

4. I'm also not fully clear on how to interpret FWHM. For example, stiffer pillars would likely dampen and broaden the signal differently than softer ones... so what exactly does a change in this metric mean in a mechanical or biological sense?

    - We thank the reviewer for this valuable remark. While pillar stiffness does influence tissue beating kinematics, the primary purpose of the FWHM metric is to quantify the duration of tissue contraction. This metric can be applied in two complementary contexts: (1) to compare tissues grown under identical pillar stiffness conditions as an indicator of contractile maturity, and (2) to compare tissues

grown under different pillar stiffnesses, enabling cross-comparison of experimental conditions and assessment of their relative effects on contractile behavior.

- We acknowledge, however, that when comparing across stiffness conditions, changes in FWHM may reflect both biological and mechanical contributions. This is an important limitation that should be considered when interpreting this metric. As recommended throughout the manuscript for all extracted metrics, FWHM should be interpreted in conjunction with other tissue-level metrics to obtain a more comprehensive and reliable characterization of contractile behavior.
- To clarify these considerations, we have added a brief discussion of the interpretation of FWHM in the "MicroBundlePillarTrack" section; the corresponding revisions are highlighted in red in the revised manuscript. We thank the reviewer for prompting us to make this important point more explicit.

5. The beginning is a bit repetitive with all the section references. It makes the writing feel heavier than it needs to be and breaks the flow a bit.

    - We appreciate this feedback and agree that the flow of the opening is important for readability. We believe the reviewer is referring to the introductory paragraph of the "Methods" section, though we warmly welcome clarification if a different passage was intended.
    - We note that the section references in this paragraph are intentional, as they provide the reader with a structured roadmap of the section's content and organization. That said, we have revised this paragraph to reduce repetition and improve readability while preserving its function, with the relevant changes highlighted in red in the revised manuscript.
    - We thank the reviewer for this helpful suggestion, which we believe has meaningfully improved the clarity of the manuscript.

6. There should be more consistency in units. Right now, pixel-based displacement isn't really comparable across systems, which seems at odds with the idea of this being broadly applicable. Some kind of normalization or conversion to physical units would help.

    - We thank the reviewer for raising this important point and agree that meaningful comparisons across systems require consistent physical units. This is a fair concern, and we are glad to have the opportunity to clarify our choices here.
    - In the present work, pixel-based displacement is justified because all image sequences in the dataset were acquired under identical imaging settings at a uniform spatial resolution of 0.908 $\mu m$/pixel. Converting pixel-based displacements to physical units is straightforward: dividing by the pixel size directly yields displacements in $\mu m$. Such a conversion would not alter any comparisons or conclusions within this dataset, as it amounts to scaling all values by a constant factor.
    - We acknowledge that for cross-platform applicability, where imaging settings and pixel sizes may differ across systems, conversion to physical units is essential to ensure meaningful comparability. The proposed framework readily supports this conversion, provided the pixel size of the imaging setup is known.
    - To address this concern and improve transparency, we have added a remark in the revised manuscript justifying the use of pixel-based units in the present work, clarifying the conversion procedure, and recommending the use of physical units for cross-platform analyses. We similarly address the use of "frames" as a temporal unit and include a corresponding note justifying this choice and describing the conversion to physical time units. The relevant changes are highlighted in red in the "Additional metrics" section. We thank the reviewer for pushing us to make these considerations explicit.

7. It would also be helpful to know how this system behaves with mature tissue. Do these metrics hold up in more mature, aligned tissues, or are they specific to earlier stages?

- We thank the reviewer for this thoughtful question. We are pleased to note that the dataset analyzed here contains tissues at varying stages of maturity, with culture ages ranging from 3 to 21 days, and the proposed metrics are not specific to any particular stage.
- While a systematic analysis of metric trends as a function of culture age and tissue maturity was not performed in the present work due to the limited number of tissues available at 21 days of culture, tracking the evolution of these metrics as tissues mature represents a meaningful and exciting direction for future work. More broadly, provided that imaging quality is sufficient to allow reliable tracking of tissue contractile displacement, the derived metrics are expected to remain meaningful and informative across maturity stages.
- To address this point, the "Conclusion" section of the revised manuscript has been updated to include metric evolution with tissue maturity as a direction for future work, with the relevant changes highlighted in red. We thank the reviewer for raising this question, as we believe it highlights an important and promising avenue for extending the framework.

8. The saddle point result is really interesting. It would be great to see more discussion on how to actually probe the biology behind it. For example, could drug perturbations, changes in tissue size, or different boundary conditions help unpack what's going on?

   - Thank you for this insightful comment. We agree that the critical point analysis yields particularly interesting results. Regarding the suggestion that changes in tissue size or boundary conditions may help unpack the underlying biology, we can say that to date we have investigated both the natural and (limited) prescribed variations throughout the dataset we had access to in tissue size, aspect ratio, curvature, and boundary conditions associated with varying pillar stiffness, and none of these factors appeared to influence the presence or location of the saddle point. However, we fully agree that it would be fascinating to approach this question in an experimentally driven way where e.g., tissue geometries were varied with this specific analysis in mind. In the original version of the manuscript, we identify several promising directions for future investigation, quoted below:

     "*Additionally, a thorough investigation of the spatial organization of critical points would be valuable– for instance, assessing whether their distribution is random or exhibits clustering within specific tissue domains. This could reveal underlying mechanical microenvironments and potential hotspots of activity. Most intriguingly, integrating critical point information with high-resolution immunostained imaging would allow for direct correlation of critical point locations with cellular architecture and tissue composition. This multimodal analysis could uncover important mechanistic links between topological features in displacement fields and the biological makeup of the tissue, paving the way for new insights into tissue structure–function relationships.*"

     In response to the reviewer's suggestion that drug perturbation studies represent a particularly promising avenue for probing the biology underlying these observations, we have added this suggestion to the future directions discussion in the revised manuscript, with the relevant changes highlighted in red in the "Critical point analysis provides complementary insights to PCA" section.

9. Typos:

   – Page 4, Section 2.2.1: "Full Width at Half Maximum" shouldn't be capitalized.

     - Thank you for this careful observation. We acknowledge that "full width at half maximum" is conventionally written in lowercase; however, we have chosen to retain the capitalized form in order to maintain consistency with our formatting convention of capitalizing all metric names throughout the manuscript. We respectfully prefer to keep this capitalization to maintain this consistency, though we would certainly be OK with removing it if the editor or reviewer felt strongly.

- Page 5, Section 2.2.2: “please refer to its original publication” → “please refer to the original publication.”
  - Thank you for this correction. The word “its” has been replaced with “the” to eliminate the ambiguity in antecedent reference, with the relevant changes highlighted in red in the revised manuscript.
- Page 28, Conclusion: “Moreover” can just be removed.
  - Thank you for this suggestion. “Moreover” has been removed.
- Page 5, Section 2.2.3: “these tissue” → “these tissues.”
  - Thank you for catching this typo. We have corrected the error and the changes are highlighted in red in the revised manuscript.
- Figure 4 caption: “disaplacement” → “displacement.”
  - Thank you for catching this typo. The spelling has been corrected in the legend of Figure 4.

---

**Reviewer 2 Comments and Response**

---

In their paper ‘Quantifying the spatiotemporal dynamics of engineered cardiac mircobundles’ by H. Kobeissi et al., the authors describe a sophisticated measurement and analysis platform for the quantification of the contractile behavior of engineered cardiac microbundles. They developed an automated analysis pipeline for characterizing the contractions of a large number of engineered heart muscle bundles, and assessed the utility of various metrics across the dataset. Overall, the work is high quality, thorough, extensive, and well done. What impresses me most is that the authors demonstrate their automated pipeline across so many tissues and provide in-depth analyses of various metrics. One of the main weaknesses of the study is that it provides only limited biological insight. At the end, the sophisticated analysis can only establish a correlation between “Tissue Mean Peak Absolute Displacement” and “Pillar Mean Peak Force,” or confirm that the “tissue contraction is primarily linear and isotropic along the major axis”. However, the authors acknowledge this issue, and, in my view, it is related to the large amounts of data, to the complexity of the biology, and the variability across so many tissues, and can therefore be excused. Many other biological studies lack the quantitative rigor of this study, and it can therefore serve as a guiding example.

1. Please add line numbers.
   - Thank you for pointing this out. We apologize for overlooking this submission guideline. We have added line numbers throughout the revised manuscript.
2. The title should be more specific: “Quantifying the spatiotemporal mechanical dynamics of engineered cardiac mircobundles” is more fitting in my opinion, because the authors quantify mechanical contraction patterns and do not measure the electrophysiology of the tissues.
   - Thank you for this suggestion. We agree that the proposed title is more specific and accurately reflects the scope of the work, and we have adopted it accordingly. The relevant change is highlighted in red in the revised manuscript.
3. Fig. 1: The left panel lacks a scale bar. Are the multiple images supposed to illustrate that each dataset is a video? Maybe add an arrow along that direction with a label ‘t’ or ‘time’. What does the displacement panel show, what are the dots and green/blue/yellow colors? There are no units/ticks on the plot axes.
   - Thank you for these constructive suggestions. We agree that the flagged elements benefit from additional clarification and have updated Fig. 1 accordingly. As this figure is intended to provide a high-level schematic overview of the study pipeline rather than to present detailed quantitative results, we have balanced implementing these changes with keeping a minimalist aesthetic.

4. Fig. 2 lacks scale bars.

   - Thank you for noting the absence of this essential figure element. We have added scale bars to the updated Fig. 2 in the revised manuscript.

5. Fig. 3: is this data from the study or are they theoretical examples illustrating the critical point analysis? Please clarify.

   - Thank you for raising this point. The figures shown in Fig. 3 are theoretical examples intended to illustrate the types of critical points, rather than data drawn from the study. We have clarified this in the caption of Fig. 3, with the relevant changes highlighted in red in the revised manuscript.

6. Fig. 7: Are these panels all from one tissue or collected from multiple tissues? As the microbundles contract, could there be changes in density and subsequent refractive index changes that alter the features being tracked?

   - We thank the reviewer for these thoughtful questions. Regarding the first question, the panels in Fig. 7 correspond to the first 10 principal components, represented as vector quiver plots, obtained from a principal component analysis performed collectively on the displacement fields of all 670 tissue samples at peak contraction. For completeness, we quote the relevant passage from the "Principal Component Analysis" subsection of the "Methods" section: "*We then concatenated the unfolded horizontal and vertical displacement matrices along the feature axis, resulting in a final data matrix* ($\mathbf{Q}$) *of size* $670 \times 1848$. *This organization allows each tissue sample to be represented as a single vector of* 1,848 *displacement features, enabling a comprehensive and meaningful principal component analysis. We performed PCA using the Python library scikit-learn (v* 1.7.1*) on the data matrix* $\mathbf{Q}$." The caption of Fig. 7 has been updated to clarify this, with the relevant changes highlighted in red in the revised manuscript.
   - Regarding the second question, we appreciate the reviewer raising this subtle but important point. Brightfield microscopy is indeed sensitive to refractive index variations, and the concern is valid in principle. However, several considerations suggest that this effect is unlikely to significantly impact the tracking results in the present work. First, the deformations undergone by hiPSC-based cardiac microbundles are relatively small, and density-driven refractive index variations within the tissue are expected to be negligible given the near-incompressibility of soft hydrated biological tissues [3]. Second, the sparse optical flow algorithm employed here tracks salient feature points, such as corners and edges, defined by strong intensity gradients. The dominant contrast at these feature points is not expected to be substantially affected by subtle refractive index variations within the tissue interior. The reliability and accuracy of the tracking under the imaging conditions of this dataset are further supported by the extensive validation studies in [1] and [2].
   - We acknowledge that for tissues undergoing larger deformations or imaged under conditions more sensitive to refractive index variations, this effect may warrant further investigation. To reflect this, a note has been added to the "Conclusion" section emphasizing that the meaningfulness of such analyses depends critically on imaging and tracking quality, with the relevant changes highlighted in red in the revised manuscript. We thank the reviewer for raising this point, as it helpfully highlights an important consideration for future applications of the framework.

7. In section 2.2.2, it is described that the tissue is tracked using "sparse optical flow," but in the next sentence, it is stated that it "yields high-resolution vector maps." Isn't this a contradiction? It would be great to mention the temporal and spatial resolution in this section and the corresponding figures.

   - We thank the reviewer for this careful observation and apologize for any confusion. While the algorithm used is sparse optical flow (in contrast to dense optical flow), the density of tracked points is kept high. Specifically, the tracking code automatically ensures a minimum coverage such that

the fiducial markers are sufficiently dense to yield high-resolution displacement vector maps, with a minimum of one fiducial marker enforced for every subdivision of 50 pixels of tissue area. Importantly, as part of the "MicroBundleCompute" software, the displacement values at these sparse marker points are subsequently interpolated across the tissue domain, yielding spatially continuous, high-resolution vector maps. It is this interpolation step that reconciles the use of sparse optical flow with the production of high-resolution displacement fields.

- As the number and spatial distribution of tracked markers vary from tissue to tissue, a single spatial resolution cannot be reported; however, Fig. 2b provides a representative illustration of the density and spatial coverage of the tracked marker points across the tissue domain. Regarding temporal resolution, tracking is performed frame by frame, and the temporal resolution is therefore determined by the frame rate at which the time-lapse sequence was acquired, which in this case is 65 Hz, as stated in the "Dataset" section.
- We have clarified the statement regarding high-resolution displacement vector maps in the "MicroBundleCompute" Section, with the relevant changes highlighted in red in the revised manuscript. We thank the reviewer for prompting us to make this distinction explicit, as we agree it is important for readers to understand how the sparse tracking and dense displacement fields relate to one another.

8. Are the fiducial markers on a regular or irregular grid? Eventually, a zoomed-in view showing a few of these markers would be very helpful to the reader.

    - We thank the reviewer for this helpful suggestion. The tracked fiducial markers have an irregular distribution that varies from tissue to tissue, both in number and spatial arrangement, as the marker points are automatically identified by the tracking software based on the Shi-Tomasi good features to track algorithm. As a brief note, this tracking approach has been validated in our prior work [1].
    - To enable meaningful cross-comparisons between tissues, the displacement results obtained on this irregular distribution of markers are interpolated onto a $28 \times 33$ regular grid, which is itself adapted to each tissue geometry to ensure consistent and comparable representations across samples. The details of this process are described in the "Spatial registration of tissue domains" section, and Fig. 2 provides an illustrative explanation of the registration procedure, showing both the original irregular distribution of tracked markers and the regular grid onto which the results are interpolated.
    - We are glad the reviewer raised this point, as it prompted us to improve the visual clarity of this aspect of the pipeline. We have updated the caption of Fig. 2 to clarify that a zoomed-in view of the markers is included, and we have added a supplementary figure (Fig. 1) in response to comment 10 below, depicting both the original irregular marker distribution and the regular interpolation grid. The relevant changes are highlighted in red in the revised manuscript. We hope these additions make the tracking procedure more transparent and accessible to the reader.

9. In the beginning of section 2.2.2.1, the authors say that they "calculate average subdomain strain rather than full-field strain". Please explain the difference (1-2 sentences).

    - We are happy to elaborate on this distinction. Average subdomain strain refers to the strain computed within a predefined subdivision of the tissue domain. Specifically, within each subdivision, the displacements of all fiducial markers are averaged, and a single representative strain value is subsequently derived from this averaged deformation. This approach therefore yields one averaged strain value per subdivision rather than a spatially resolved strain estimate at each marker location. To provide a concrete illustration, if the tissue is divided into a $3 \times 5$ grid, a total of 15 subdivisions are obtained, each characterized by a single averaged strain value. Full-field strain, by contrast, refers to the strain estimated individually at each fiducial marker point via the method described in the "Strain computation details" section.

- We have added a brief clarification of this distinction in the "Strain computation details" section of the revised manuscript, with the relevant changes highlighted in red. We thank the reviewer for flagging this, as we agree that making this distinction explicit improves the clarity of the methods description.

10. Section 2.2.2.1 requires more visuals or drawings to illustrate the strain computations. It would be great to see a specific example of how strain is computed from displacements and overlaid onto parts of the microbundle.

    - Thank you for this constructive suggestion. We have added a supplementary figure (Fig. 1) illustrating the strain computation procedure, along with a descriptive caption. This figure is referenced in Section 2.2.2.1 and at other relevant points in the manuscript where visual clarification is beneficial. The relevant changes are highlighted in red in the revised manuscript.

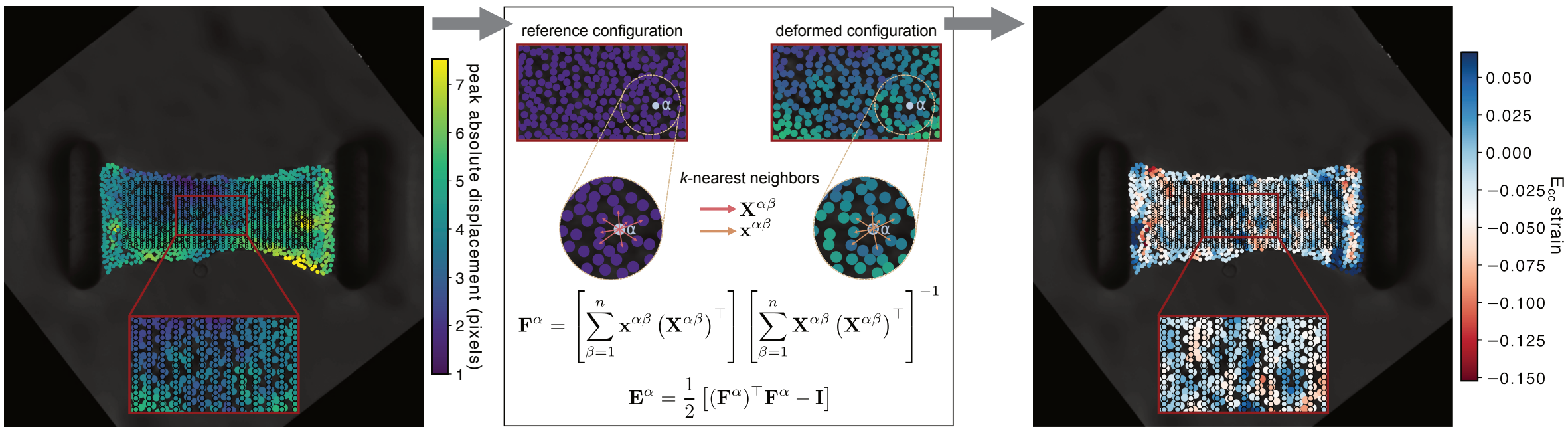


Figure 1: Details of computing a Green-Lagrange strain field from tracked displacement data. The left panel shows the absolute displacement field at peak contraction, obtained at the tracked fiducial marker points and interpolated onto a $28 \times 33$ regular grid; the zoomed-in inset highlights the spatial distribution of fiducial marker points within the tissue domain. Directional displacement fields in the column (horizontal) and row (vertical) directions are obtained analogously. The locations of the tracked fiducial marker points in both the reference (undeformed) and current (deformed) configurations serve as the input to the strain computation illustrated in the middle panel. There, a $k$-nearest neighbor approach is used to estimate the local deformation gradient at each fiducial marker point. For a given marker point $\alpha$ (here shown with $k = 8$ neighbors), the vectors $\mathbf{X}^{\alpha\beta}$ and $\mathbf{x}^{\alpha\beta}$ connect point $\alpha$ to each neighbor $\beta$ in the reference and current configurations, respectively. The deformation gradient tensor $\mathbf{F}^{\alpha}$ is estimated from these neighbor vectors via a least-squares fitting procedure, and the Green-Lagrange strain tensor $\mathbf{E}^{\alpha}$ is subsequently computed as shown. Repeating this procedure at every fiducial marker point yields the full-field strain estimate shown in the right panel, where the Green-Lagrange strain in the column-column direction $E_{cc}$ is displayed at all fiducial marker points and interpolated onto the $28 \times 33$ regular grid. Positive and negative values indicate local extension and compression, respectively, as indicated by the color scale.

11. In section 2.2.2.2, the authors mention that some movies had to be excluded, because of blurring. What else ensures that the fiducial markers can be identified properly? Is it back-illumination? This should be mentioned somewhere in the manuscript.

    - We thank the reviewer for this thoughtful suggestion. In the "Software failure and data exclusion" section, movies were excluded for two main reasons: blurred image frames and recordings containing fewer than three full contractions. We acknowledge that other imaging-related factors could in principle lead to unusable recordings; for instance, insufficient back-illumination leading to poor image contrast, or excessive tissue movement outside the field of view, could similarly render recordings unsuitable for analysis.

    - While none of these additional factors were encountered in the present dataset, we agree that acknowledging them improves the completeness of this section. We have updated the "Software failure and data exclusion" section accordingly, with the relevant changes highlighted in red in the revised

manuscript. We are grateful to the reviewer for prompting this addition, as we believe it makes the discussion of data quality and exclusion criteria more thorough and informative.

12. On p.6, the authors describe the transformation of the data into a 3D matrix with dimensions 28 x 33 x 670 matrices before PCA analysis. The first and second dimensions are the horizontal and vertical dimensions of the field, and the third is the number of samples. What about the x and y components of the displacement vectors?

    - We thank the reviewer for this careful observation and are happy to clarify. As stated in the manuscript, two separate three-dimensional matrices of dimensions $28 \times 33 \times 670$ are constructed: one for the horizontal (x) components and one for the vertical (y) components of the displacement field. For convenience, we quote the relevant passage directly: "*To organize the data for PCA, we first retained the spatial structure of the grid points and constructed* ***two three-dimensional matrices of size*** $\mathbf{28 \times 33 \times 670}$***, one for the horizontal (column) and one for the vertical (row) components of the displacement field.***"
    - To perform PCA, these two matrices are subsequently unfolded into two-dimensional matrices and concatenated along the feature axis, ensuring that both displacement components are jointly accounted for in the analysis. The details of this process are described in the "Principal Component Analysis" section of the manuscript, which has been revised to further clarify this point, with the relevant changes highlighted in red. We thank the reviewer for prompting us to make this clearer, as we agree that the treatment of the vector components is an important detail for readers to follow.

13. The critical point analysis appears to be adopted from fluid dynamics. Is this a good approach? Aren't fluid dynamics and mechanics two very different physical phenomena?

    - We thank the reviewer for raising this important question. Critical point analysis is indeed most commonly encountered in the context of fluid dynamics, so the question of its applicability to mechanical systems is a very natural one. The key insight is that the method is rooted in vector field topology and dynamical systems theory, which are mathematical frameworks that are not tied to any specific physical phenomenon. As such, the method can be meaningfully applied to any continuous vector field, including the displacement fields characterizing cardiac tissue contraction.
    - We do address this point in the manuscript in the "Critical point analysis" section, where we note that "*the versatility of vector field analysis and critical point identification has captured the interest of researchers across a broad range of disciplines, given its applicability to virtually any vector field.*" This statement is further supported by examples of critical point analysis applied across a range of fields beyond fluid dynamics, as cited in that section.
    - We have added a brief clarifying sentence to the manuscript to make this rationale more explicit upfront, with the relevant changes highlighted in red. We are glad the reviewer raised this question, as we agree that clearly motivating the use of a technique borrowed from another field is important for readers who may be less familiar with its mathematical foundations.

14. The authors mention in section 3.3 that the critical point analysis might be used to "reveal mechanical microenvironments or hotspots of activity" and uncover "links between topological features in displacement fields and the biological makeup of the tissue". What do they mean by that?

    - We thank the reviewer for pointing this out and are happy to elaborate. By "mechanical microenvironments," we refer to spatially distinct regions within the tissue that experience different local mechanical conditions, arising from variations in tissue composition and organization such as matrix stiffness, fiber alignment, or sarcomere organization. By "hotspots of activity," we refer to regions where significant mechanical events are concentrated, such as areas of locally elevated deformation or

strain. Critical points, being locations where the displacement vector vanishes, may delineate boundaries between such regions or coincide with such hotspots, and we believe they carry meaningful information about the local mechanical state of the tissue.

- Regarding "links between topological features and the biological makeup of the tissue," we refer to the potential co-localization of critical points with specific biological structural features. This could be investigated in future work by integrating critical point information with fluorescence imaging to directly correlate these topological features with the underlying cellular architecture and tissue composition.
- We have added a brief clarification of these concepts in the revised manuscript, with the relevant changes highlighted in red in the "Interpretable features show continuous variation across experimental conditions" section. We appreciate the reviewer pushing us to be more precise here, as we agree these are exciting but speculative ideas that deserve clearer framing.

15. Did the authors use this analysis to examine how contractile patterns change longitudinally over time? What would the authors hope to learn from such an analysis?

    - We thank the reviewer for this thoughtful question, and we agree that longitudinal analysis of contractile patterns is a compelling direction. Unfortunately, a longitudinal analysis was not performed in the present work due to limitations in the available dataset, which was originally designed by our collaborators to address specific research questions in their study rather than to support longitudinal tracking. Although the dataset spans culture ages from 3 to 21 days, the sample distribution across time points is highly uneven: the majority of tissues are from day 7, only two sets of experimental conditions include measurements at both day 3 and day 21, and the number of surviving tissues at day 21 is significantly lower than at day 3, rendering a direct longitudinal comparison statistically unreliable.
    - That said, this is precisely the type of analysis we hope to pursue with this framework in the future. A well-powered longitudinal study with balanced sample numbers across multiple time points could enable the use of these metrics as maturity indicators, capturing the progression toward a stable contractile phenotype and potentially revealing how topological and kinematic features reorganize as tissues develop.
    - We have added a note to this effect in the future directions discussion in the "Conclusion" section, with the relevant changes highlighted in red. We thank the reviewer for raising this point, as it highlights one of the most exciting potential applications of the framework going forward.

16. The study mentions other related work within the microbundle tracking field, but largely ignores other adjacent work in which tracking and motion analysis are performed with fluorescence-based imaging data. Since the authors mention calcium imaging in the Discussion, they should consider mentioning work by:

    – J. Lebert et al., Real-time optical mapping of contracting cardiac tissues with GPU-accelerated numerical motion tracking, Front. Cardiovasc. Med., 2022. This study demonstrates numerical tracking and measurement of action potential waves in whole hearts and engineered cardiac tissues. It also compares different optical flow-based tracking algorithms.

    – optimap: An open-source library for processing fluorescence video data: `https://github.com/cardiacvision/optimap/`

    – L. Woodhams et al., Virtual blebbistatin: A robust and rapid software approach to motion artifact removal in optical mapping of cardiomyocytes, PNAS, 2023. This study discusses strain computations and simultaneous electrophysiology measurements in engineered cardiac tissue.

- We thank the reviewer for these helpful suggestions and agree that better situating our contribution within the broader landscape of motion tracking and analysis in fluorescence-based cardiac imaging will strengthen the manuscript.
- The primary focus of the present study is the analysis of contractile dynamics in engineered heart tissues and cardiac microbundles using brightfield and phase-contrast imaging. Accordingly, the literature reviewed in the "Introduction" was selected to emphasize methods developed for engineered tissue platforms and label-free imaging modalities most closely related to the intended application of our pipeline. That said, we fully agree that highlighting relevant developments from adjacent imaging domains provides valuable context for the reader.
- In response to the reviewer's suggestion, we have incorporated citations to Lebert et al. (2022) and Woodhams et al. (2023) into the "Introduction" section, with the relevant changes highlighted in red.

# References

[1] Kobeissi H, Jilberto J, Karakan MÇ, Gao X, DePalma SJ, Das SL, et al. MicroBundleCompute: Automated segmentation, tracking, and analysis of subdomain deformation in cardiac microbundles. Plos one. 2024;19(3):e0298863. PMID: 38530829.

[2] Kobeissi H, Gao X, DePalma SJ, Ewoldt JK, Wang MC, Das SL, et al. MicroBundlePillarTrack: A Python package for automated segmentation, tracking, and analysis of pillar deflection in cardiac microbundles. Micropublication Biology. 2024;2024. PMID: 39114859.

[3] Fung Yc. Chapter 10: Heart Muscle. In: Biomechanics: mechanical properties of living tissues. Springer Science & Business Media; 2013. .
