# Supplementary material for: Quantifying the spatiotemporal mechanical dynamics of engineered cardiac microbundles
Source: PLoS Comput Biol. 2026 Jul 20;22(7):e1014522. doi: 10.1371/journal.pcbi.1014522 (PMC13492994; doi:10.1371/journal.pcbi.1014522)
Supplement: Peer review history

# Peer review: Decision Letter 0

**DOI:** 10.1371/journal.pcbi.1014522.r001

**Authors:** Dimitrios Vavylonis, Anna Grosberg

5 May 2026

PCOMPBIOL-D-26-00827

Quantifying the Spatiotemporal Dynamics of Engineered Cardiac Microbundles

PLOS Computational Biology

Dear Dr. Lejeune,

Thank you for submitting your manuscript to PLOS Computational Biology. After careful consideration, we feel that it has merit but does not fully meet PLOS Computational Biology's publication criteria as it currently stands. Therefore, we invite you to submit a revised version of the manuscript that addresses the points raised during the review process. Please make sure to address all the concerns raised by the reviewers with particular emphasis to ensuring consistency of units and clarifying the biological conclusions.

Please submit your revised manuscript by Jul 05 2026 11:59PM. If you will need more time than this to complete your revisions, please reply to this message or contact the journal office at ploscompbiol@plos.org. When you're ready to submit your revision, log on to https://www.editorialmanager.com/pcompbiol/ and select the 'Submissions Needing Revision' folder to locate your manuscript file.

Please include the following items when submitting your revised manuscript:

* A letter that responds to each point raised by the editor and reviewer(s). You should upload this letter as a separate file labeled 'Response to Reviewers'. This file does not need to include responses to formatting updates and technical items listed in the 'Journal Requirements' section below.

* A marked-up copy of your manuscript that highlights changes made to the original version. You should upload this as a separate file labeled 'Revised Manuscript with Track Changes'.

* An unmarked version of your revised paper without tracked changes. You should upload this as a separate file labeled 'Manuscript'.

If you would like to make changes to your financial disclosure, competing interests statement, or data availability statement, please make these updates within the submission form at the time of resubmission. Guidelines for resubmitting your figure files are available below the reviewer comments at the end of this letter.

As the corresponding author, your ORCID iD is verified in the submission system and will appear in the published article. PLOS supports the use of ORCID, and we encourage all coauthors to register for an ORCID iD and use it as well. Please encourage your coauthors to verify their ORCID iD within the submission system before final acceptance, as unverified ORCID iDs will not appear in the published article. Only the individual author can complete the verification step; PLOS staff cannot verify ORCID iDs on behalf of authors.

We look forward to receiving your revised manuscript.

Kind regards,

Anna Grosberg, Ph.D.

Academic Editor

PLOS Computational Biology

Dimitrios Vavylonis

Section Editor

PLOS Computational Biology

**Journal Requirements:**

If the reviewer comments include a recommendation to cite specific previously published works, please review and evaluate these publications to determine whether they are relevant and should be cited. There is no requirement to cite these works unless the editor has indicated otherwise.

1) Please ensure that the CRediT author contributions listed for every co-author are completed accurately and in full.

At this stage, the following Authors/Authors require contributions: Brendon M. Baker, Samuel J. DePalma, Javiera Jilberto, Hiba Kobeissi, David Nordsletten, and Emma Lejeune. Please ensure that the full contributions of each author are acknowledged in the "Add/Edit/Remove Authors" section of our submission form.

The list of CRediT author contributions may be found here: https://journals.plos.org/ploscompbiol/s/authorship#loc-author-contributions

2) We ask that a manuscript source file is provided at Revision. Please upload your manuscript file as a .doc, .docx, .rtf or .tex. If you are providing a .tex file, please upload it under the item type u2018LaTeX Source Fileu2019 and leave your .pdf version as the item type u2018Manuscriptu2019.

3) Your manuscript's sections are not in the correct order.  Please amend to the following order: Abstract, Introduction, Results, Discussion, and Methods

4) Please upload all main figures as separate Figure files in .tif or .eps format. For more information about how to convert and format your figure files please see our guidelines:

https://journals.plos.org/ploscompbiol/s/figures

5) We notice that your supplementary Figures are included in the manuscript file. Please remove them and upload them with the file type 'Supporting Information'. Please ensure that each Supporting Information file has a legend listed in the manuscript after the references list.

6) Please amend your detailed Financial Disclosure statement. This is published with the article. It must therefore be completed in full sentences and contain the exact wording you wish to be published.

1) State the initials, alongside each funding source, of each author to receive each grant. For example: "This work was supported by the National Institutes of Health (####### to AM; ###### to CJ) and the National Science Foundation (###### to AM)."

2) State what role the funders took in the study. If the funders had no role in your study, please state: "The funders had no role in study design, data collection and analysis, decision to publish, or preparation of the manuscript."

3) If any authors received a salary from any of your funders, please state which authors and which funders..

If you did not receive any funding for this study, please simply state: u201cThe authors received no specific funding for this work.u201d

**Reviewers' comments:**

Reviewer's Responses to Questions

**Comments to the Authors:**

**Please note here if the review is uploaded as an attachment.**

Reviewer #1: Comments:

It’s not really clear how this method translates from 2D to 3D systems. Has there been any validation to show that what’s measured in 2D holds up in more physiologically relevant 3D tissues?

The scalability across different tissue platforms feels underdeveloped. Tissue sizes vary a lot, and so do imaging setups. Right now, it’s not clear at what resolution this method starts to break down. There’s no guidance on minimum pixel size, no sensitivity analysis for resolution, and no real discussion of how tracking accuracy changes as resolution drops.

FWHM is introduced without explanation (Section 2.2.1) and only defined later. It would help to define it right when it first appears.

I’m also not fully clear on how to interpret FWHM. For example, stiffer pillars would likely dampen and broaden the signal differently than softer ones... so what exactly does a change in this metric mean in a mechanical or biological sense?

The beginning is a bit repetitive with all the section references. It makes the writing feel heavier than it needs to be and breaks the flow a bit.

There should be more consistency in units. Right now, pixel-based displacement isn’t really comparable across systems, which seems at odds with the idea of this being broadly applicable. Some kind of normalization or conversion to physical units would help.

It would also be helpful to know how this system behaves with mature tissue. Do these metrics hold up in more mature, aligned tissues, or are they specific to earlier stages?

The saddle point result is really interesting. It would be great to see more discussion on how to actually probe the biology behind it. For example, could drug perturbations, changes in tissue size, or different boundary conditions help unpack what’s going on?

Typos:

Page 4, Section 2.2.1: “Full Width at Half Maximum” shouldn’t be capitalized.

Page 5, Section 2.2.2: “please refer to its original publication” → “please refer to the original publication.”

Page 28, Conclusion: “Moreover” can just be removed.

Page 5, Section 2.2.3: “these tissue” → “these tissues.”

Figure 4 caption: “disaplacement” → “displacement.”

Reviewer #2: In their paper ‘Quantifying the spatiotemporal dynamics of engineered cardiac mircobundles” by H. Kobeissi et al., the authors describe a sophisticated measurement and analysis platform for the quantification of the contractile behavior of engineered cardiac microbundles. They developed an automated analysis pipeline for characterizing the contractions of a large number of engineered heart muscle bundles, and assessed the utility of various metrics across the dataset. Overall, the work is high quality, thorough, extensive, and well done. What impresses me most is that the authors demonstrate their automated pipeline across so many tissues and provide in-depth analyses of various metrics. One of the main weaknesses of the study is that it provides only limited biological insight. At the end, the sophisticated analysis can only establish a correlation between “Tissue Mean Peak Absolute Displacement” and “Pillar Mean Peak Force,” or confirm that the “tissue contraction is primarily linear and isotropic along the major axis”. However, the authors acknowledge this issue, and, in my view, it is related to the large amounts of data, to the complexity of the biology, and the variability across so many tissues, and can therefore be excused. Many other biological studies lack the quantitative rigor of this study, and it can therefore serve as a guiding example.

Comments:

Please add line numbers.

The title should be more specific: “Quantifying the spatiotemporal mechanical dynamics of engineered cardiac mircobundles” is more fitting in my opinion, because the authors quantify mechanical contraction patterns and do not measure the electrophysiology of the tissues.

Fig. 1: The left panel lacks a scale bar. Are the multiple images supposed to illustrate that each dataset is a video? Maybe add an arrow along that direction with a label ’t’ or ‘time’.What does the displacement panel show, what are the dots and green/blue/yellow colors? There are no units/ticks on the plot axes.

Fig. 2 lacks scale bars.

Fig. 3: is this data from the study or are they theoretical examples illustrating the critical point analysis? Please clarify.

Fig. 7: Are these panels all from one tissue or collected from multiple tissues?

As the microbundles contract, could there be changes in density and subsequent refractive index changes that alter the features being tracked?

In section 2.2.2, it is described that the tissue is tracked using “sparse optical flow,” but in the next sentence, it is stated that it “yields high-resolution vector maps.” Isn’t this a contradiction? It would be great to mention the temporal and spatial resolution in this section and the corresponding figures.

Are the fiducial markers on a regular or irregular grid? Eventually, a zoomed-in view showing a few of these markers would be very helpful to the reader.

In the beginning of section 2.2.2.1, the authors say that they “calculate average subdomain strain rather than full-field strain”. Please explain the difference (1-2 sentences).

Section 2.2.2.1 requires more visuals or drawings to illustrate the strain computations. It would be great to see a specific example of how strain is computed from displacements and overlaid onto parts of the microbundle.

In section 2.2.2.2, the authors mention that some movies had to be excluded, because of blurring. What else ensures that the fiducial markers can be identified properly? Is it back-illumination? This should be mentioned somewhere in the manuscript.

On p.6, the authors describe the transformation of the data into a 3D matrix with dimensions 28 x 33 x 670 matrices before PCA analysis. The first and second dimensions are the horizontal and vertical dimensions of the field, and the third is the number of samples. What about the x and y components of the displacement vectors?

The critical point analysis appears to be adopted from fluid dynamics. Is this a good approach? Aren’t fluid dynamics and mechanics two very different physical phenomena?

The authors mention in section 3.3 that the critical point analysis might be used to “reveal mechanical microenvironments or hotspots of activity” and uncover “links between topological features in displacement fields and the biological makeup of the tissue”. What do they mean by that?

Did the authors use this analysis to examine how contractile patterns change longitudinally over time? What would the authors hope to learn from such an analysis?

The study mentions other related work within the microbundle tracking field, but largely ignores other adjacent work in which tracking and motion analysis are performed with fluorescence-based imaging data. Since the authors mention calcium imaging in the Discussion, they should consider mentioning work by:

- J. Lebert et al., Real-time optical mapping of contracting cardiac tissues with GPU-accelerated numerical motion tracking, Front. Cardiovasc. Med., 2022. This study demonstrates numerical tracking and measurement of action potential waves in whole hearts and engineered cardiac tissues. It also compares different optical flow-based tracking algorithms.

- optimap: An open-source library for processing fluorescence video data: https://github.com/cardiacvision/optimap/

- L .Woodhams et al., Virtual blebbistatin: A robust and rapid software approach to motion artifact removal in optical mapping of cardiomyocytes, PNAS, 2023. This study discusses strain computations and simultaneous electrophysiology measurements in engineered cardiac tissue.

**********

**Have the authors made all data and (if applicable) computational code underlying the findings in their manuscript fully available?**

The PLOS Data policy requires authors to make all data and code underlying the findings described in their manuscript fully available without restriction, with rare exception (please refer to the Data Availability Statement in the manuscript PDF file). The data and code should be provided as part of the manuscript or its supporting information, or deposited to a public repository. For example, in addition to summary statistics, the data points behind means, medians and variance measures should be available. If there are restrictions on publicly sharing data or code —e.g. participant privacy or use of data from a third party—those must be specified.

Reviewer #1: Yes

Reviewer #2: None

**********

PLOS authors have the option to publish the peer review history of their article (what does this mean?). If published, this will include your full peer review and any attached files.

If you choose “no”, your identity will remain anonymous but your review may still be made public.

**Do you want your identity to be public for this peer review?**  For information about this choice, including consent withdrawal, please see our Privacy Policy.

Reviewer #1: No

Reviewer #2: No

**Figure resubmission:**

After uploading your figures to PLOS’s NAAS tool - https://ngplosjournals.pagemajik.ai/artanalysis, NAAS will process the files provided and display the results in the "Uploaded Files" section of the page as the processing is complete. If the uploaded figures meet our requirements (or NAAS is able to fix the files to meet our requirements), the figure will be marked as "fixed" above. If NAAS is unable to fix the files, a red "failed" label will appear above. When NAAS has confirmed that the figure files meet our requirements, please download the file via the download option, and include these NAAS processed figure files when submitting your revised manuscript.

**Reproducibility:**

To enhance the reproducibility of your results, we recommend that authors of applicable studies deposit laboratory protocols in protocols.io, where a protocol can be assigned its own identifier (DOI) such that it can be cited independently in the future. Additionally, PLOS ONE offers an option to publish peer-reviewed clinical study protocols. Read more information on sharing protocols at https://plos.org/protocols?utm_medium=editorial-email&utm_source=authorletters&utm_campaign=protocols

# Author comment: Author response to Decision Letter 1

**DOI:** 10.1371/journal.pcbi.1014522.r002

15 Jun 2026

Attachment Submitted filename: rebuttal.pdf

# Peer review: Decision Letter 1

**DOI:** 10.1371/journal.pcbi.1014522.r003

**Authors:** Dimitrios Vavylonis, Anna Grosberg

1 Jul 2026

Dear Dr. Lejeune,

We are pleased to inform you that your manuscript 'Quantifying the Spatiotemporal Mechanical Dynamics of Engineered Cardiac Microbundles' has been provisionally accepted for publication in PLOS Computational Biology.

Before your manuscript can be formally accepted you will need to complete some formatting changes, which you will receive in a follow up email. A member of our team will be in touch with a set of requests.

Please note that your manuscript will not be scheduled for publication until you have made the required changes, so a swift response is appreciated.

IMPORTANT: The editorial review process is now complete. PLOS will only permit corrections to spelling, formatting or significant scientific errors from this point onwards. Requests for major changes, or any which affect the scientific understanding of your work, will cause delays to the publication date of your manuscript.

Should you, your institution's press office or the journal office choose to press release your paper, you will automatically be opted out of early publication. We ask that you notify us now if you or your institution is planning to press release the article. All press must be co-ordinated with PLOS.

Thank you again for supporting Open Access publishing; we are looking forward to publishing your work in PLOS Computational Biology.

Best regards,

Anna Grosberg, Ph.D.

Academic Editor

PLOS Computational Biology

Dimitrios Vavylonis

Section Editor

PLOS Computational Biology

***********************************************************

Reviewer's Responses to Questions

**Comments to the Authors:**

**Please note here if the review is uploaded as an attachment.**

Reviewer #1: The authors have adequately addressed our comments, and we have no further substantive concerns.

Reviewer #2: I would like to thank the authors for their in-depth responses and for carefully revising their paper. All my comments and concerns were sufficiently addressed.

**********

**Have the authors made all data and (if applicable) computational code underlying the findings in their manuscript fully available?**

The PLOS Data policy requires authors to make all data and code underlying the findings described in their manuscript fully available without restriction, with rare exception (please refer to the Data Availability Statement in the manuscript PDF file). The data and code should be provided as part of the manuscript or its supporting information, or deposited to a public repository. For example, in addition to summary statistics, the data points behind means, medians and variance measures should be available. If there are restrictions on publicly sharing data or code —e.g. participant privacy or use of data from a third party—those must be specified.

Reviewer #1: None

Reviewer #2: Yes

**********

PLOS authors have the option to publish the peer review history of their article (what does this mean?). If published, this will include your full peer review and any attached files.

If you choose “no”, your identity will remain anonymous but your review may still be made public.

**Do you want your identity to be public for this peer review?**  For information about this choice, including consent withdrawal, please see our Privacy Policy.

Reviewer #1: No

Reviewer #2: No

# Editor report: Acceptance letter

**DOI:** 10.1371/journal.pcbi.1014522.r004

**Authors:** Dimitrios Vavylonis, Anna Grosberg

PCOMPBIOL-D-26-00827R1

Quantifying the Spatiotemporal Mechanical Dynamics of Engineered Cardiac Microbundles

Dear Dr Lejeune,

I am pleased to inform you that your manuscript has been formally accepted for publication in PLOS Computational Biology. Your manuscript is now with our production department and you will be notified of the publication date in due course.

The corresponding author will soon be receiving a typeset proof for review, to ensure errors have not been introduced during production. Please review the PDF proof of your manuscript carefully, as this is the last chance to correct any errors. Please note that major changes, or those which affect the scientific understanding of the work, will likely cause delays to the publication date of your manuscript.

Soon after your final files are uploaded, unless you have opted out, the early version of your manuscript will be published online. The date of the early version will be your article's publication date. The final article will be published to the same URL, and all versions of the paper will be accessible to readers.

For Research, Software, and Methods articles, you will receive an invoice from PLOS for your publication fee after your manuscript has reached the completed accept phase. If you receive an email requesting payment before acceptance or for any other service, this may be a phishing scheme. Learn how to identify phishing emails and protect your accounts at https://explore.plos.org/phishing.

Thank you again for supporting PLOS Computational Biology and open-access publishing. We are looking forward to publishing your work!

With kind regards,

Sharmila Kamatchi

PLOS Computational Biology | Carlyle House, Carlyle Road, Cambridge CB4 3DN | United Kingdom ploscompbiol@plos.org | Phone +44 (0) 1223-442824 | ploscompbiol.org | @PLOSCompBiol
